# Rapid Cis–Trans Coevolution Driven by a Novel Gene Retroposed from a Eukaryotic Conserved CCR4–NOT Component in *Drosophila*

**DOI:** 10.3390/genes13010057

**Published:** 2021-12-26

**Authors:** Benjamin H. Krinsky, Robert K. Arthur, Shengqian Xia, Dylan Sosa, Deanna Arsala, Kevin P. White, Manyuan Long

**Affiliations:** 1 Committee on Evolutionary Biology, University of Chicago, Chicago, IL 60637, USA; bhkrinsky@gmail.com; 2 Department of Ecology and Evolution, University of Chicago, Chicago, IL 60637, USA; robertkarthur@gmail.com (R.K.A.); shengqianxia@uchicago.edu (S.X.); dylansosa@uchicago.edu (D.S.); arsala@uchicago.edu (D.A.); kevin@tempus.com (K.P.W.); 3 Institute for Genomics and Systems Biology, Department of Human Genetics, University of Chicago and Argonne National Laboratory, Chicago, IL 60637, USA

**Keywords:** novel gene, driven force, cis–trans coevolution, DNA motif coevolution, ChIP-Seq, *Caf40*, *Zeus*, differentially expressed genes (DEGs), CCR4–NOT

## Abstract

Young, or newly evolved, genes arise ubiquitously across the tree of life, and they can rapidly acquire novel functions that influence a diverse array of biological processes. Previous work identified a young regulatory duplicate gene in *Drosophila*, *Zeus* that unexpectedly diverged rapidly from its parent, *Caf40*, an extremely conserved component in the CCR4–NOT machinery in post-transcriptional and post-translational regulation of eukaryotic cells, and took on roles in the male reproductive system. This neofunctionalization was accompanied by differential binding of the Zeus protein to loci throughout the *Drosophila* *melanogaster* genome. However, the way in which new DNA-binding proteins acquire and coevolve with their targets in the genome is not understood. Here, by comparing *Zeus* ChIP-Seq data from *D. melanogaster* and *D. simulans* to the ancestral Caf40 binding events from *D. yakuba*, a species that diverged before the duplication event, we found a dynamic pattern in which Zeus binding rapidly coevolved with a previously unknown DNA motif, which we term Caf40 and Zeus-Associated Motif (CAZAM), under the influence of positive selection. Interestingly, while both copies of *Zeus* acquired targets at male-biased and testis-specific genes, *D. melanogaster* and *D. simulans* proteins have specialized binding on different chromosomes, a pattern echoed in the evolution of the associated motif. Using CRISPR-Cas9-mediated gene knockout of *Zeus* and RNA-Seq, we found that *Zeus* regulated the expression of 661 differentially expressed genes (DEGs). Our results suggest that the evolution of young regulatory genes can be coupled to substantial rewiring of the transcriptional networks into which they integrate, even over short evolutionary timescales. Our results thus uncover dynamic genome-wide evolutionary processes associated with new genes.

## 1. Introduction

The origin of new genes can lead to the evolution of new and crucial functions in various biological processes, including gene regulation [1,2,3]. Regulatory and other putative functional elements can now be investigated on a genome-wide scale in order to systematically characterize networks of gene–gene interactions [4,5] and to compare patterns of conservation and divergence of gene regulation in multiple closely related species [6,7,8]. However, most of the comparisons to date have focused on conserved factors with well-characterized molecular functions [6,9]. Thus, there exists a unique opportunity to apply these approaches to investigate the evolution of new regulatory genes, as well as their effects on bound regulatory elements, and therefore explore how newly arisen loci might evolve altered or gene–gene interactions across the genome.

Retrogene movement within and between chromosomes plays a remarkable role in contributing to genetic novelty [10,11,12]. Case studies suggest that those retrogenes rapidly evolved essential developmental function [5,13]. The autosome gene *Zeus* (*CG9573*, also known as *Rcd-1r*, required for cell differentiation 1 related) is a testis-specific young gene that arose via a “out of X” retroposition event approximately 5 million years ago in the lineage, leading to *Drosophila melanogaster* and its closest relatives. *Zeus* subsequently underwent a very rapid period of molecular evolution [14,15]. Functional analyses suggest that *Zeus* evolved specific roles in the development and function of *Drosophila* sperm and testis [16]. This evolution in *Zeus*’s function coincided with changes in its expression and patterns of histone modification at the Zeus locus [17]. 

In contrast, its parental gene *Caf40* (*CG14213*, also known as *Rcd-1*), conserved across eukaryotes, is ubiquitously expressed and is essential for viability in *D. melanogaster* [16]. On the molecular level, it had been previously inferred that Caf40 has nucleic acid–binding properties and, thus, might act as a regulator through its interactions with genomic DNA [18]. By performing chromatin immunoprecipitation, followed by microarray analysis (ChIP-chip), it was subsequently discovered that both Zeus and Caf40 from *D. melanogaster* bind to several hundred sites throughout the genome, and that Zeus has acquired a number of novel regulatory targets in the genome, as is consistent with neofunctionalization following duplication [16]. 

**Figure 1:**
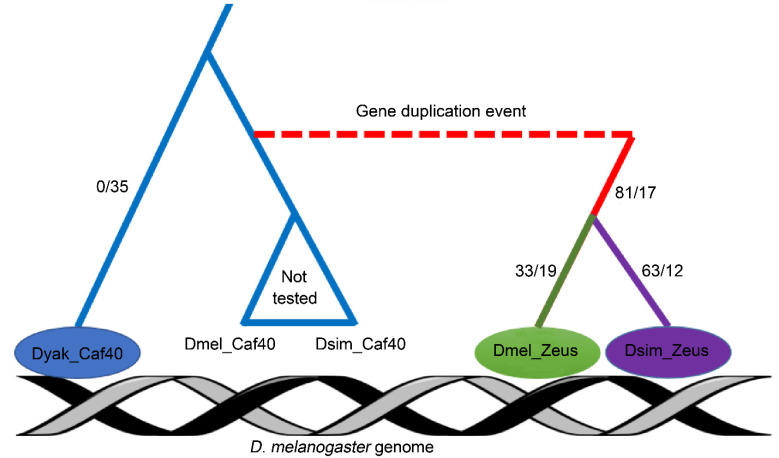
Design and results of ChIP-Seq experiments. Depiction of *Zeus/Caf40* phylogeny, with experimental design. *Zeus* originated from a gene duplication event 4–6 million years ago, before the split of *D. melanogaster* and *D. yakuba*. We sampled two copies of *Zeus* (*D. melanogaster* and *D. simulans*), as well as a single copy of *Caf40* from *D. yakuba*, which represents the ancestral pre-duplication state of the protein. All three proteins were introduced into the *D. melanogaster* genome with 3x FLAG tags attached in order to eliminate problems with variable antibody affinity. Numbers indicate the volume of nonsynonymous (before the slash) and synonymous (after the slash) changes.

The *Zeus* locus arose after the divergence of the lineages that led to *D. melanogaster* and *D. yakuba*, but prior to the divergence of *D. melanogaster* and one of its closest sister species, *D. simulans*. The Zeus protein subsequently acquired a large number of species-specific substitutions along these two lineages [16] (Figure 1). To understand patterns of lineage specific regulatory evolution of *Zeus*, as well as its initial divergence from the ancestral state of *Caf40*, we have characterized the genome-wide binding profiles of Zeus from *D. melanogaster* and its sister species *D. simulans*, as well as Caf40 from *D. yakuba* by using chromatin immunoprecipitation, followed by ChIP-Seq. We elected *D. yakuba* (pre-duplication) Caf40 as the best proxy from which to infer ancestral Caf40 binding, because the *D. yakuba* and *D. melanogaster* proteins differ in only four positions. Lastly, we employed CRISPR-Cas9 to delete *Zeus* in *D. melanogaster* and transcriptional profiling to identify CAZAM (Caf40 and Zeus-Associated Motif)-containing genes whose expression is dependent on Zeus. 

## 2. Materials and Methods

### 2.1. piggyBac Vector Construction

For *D. simulans Zeus* (*Dsim\GD22367*) and *D. melanogaster Zeus* (*CG9573*), genomic DNA was isolated from strains w501 and w1118, respectively (5 adult flies/each), using the Quick gDNA Miniprep kit (Zymo Research). Each *Zeus* coding region (CDS) was then amplified by PCR, using the iProof high-fidelity master mix (Bio-Rad Laboratories, Inc.). Primer sequences are as follows: 

Dsim_Zeus forward primer: 5′- CACCATGAGTGAGGAACCAATTCCG-3′; 

Dsim_Zeus reverse primer: 5′-CTAGGAGCCCTCTGTCGACTC-3′; 

Dmel_Zeus forward primer: 5′-CACCATGAGTGCGGAACCAAGTC-3′; 

Dmel_Zeus reverse primer: 5′-CTAGGAGGAGCCCATTGG-3′. 

The following PCR conditions were used for *D. simulans Zeus*: 98 °C for 30 s, followed by 35 cycles 98 °C for 10 s, 63 °C for 20 s and 72 °C for 15 s, followed by a final extension at 72 °C for 10 min. To amplify *D. melanogaster Zeus*, identical PCR conditions were used, except that a 61 °C annealing temperature was used.

For *D. yakuba CAF40* (*Dyak\GE15860*), total RNA was isolated from a spontaneous *ebony*-*white* mutant strain derived from the stock Täi18 (flies provided by Dr. Daniel Matute, University of North Carolina), using the RNeasy Mini Kit (Qiagen) from 5 adult flies. From the RNA sample, cDNA was synthesized by using the SuperScript III Reverse Transcriptase kit (Invitrogen) with oligo(dT). High-fidelity PCR for *CAF40* was then carried out, as it was above, using *D. yakuba* cDNA as the template; primer sequences are as follows: 

Dyak_Caf40 forward primer: 5′-CACCATGAGTGCGCAACCAAGTC-3′; 

Dyak_Caf40 reverse primer: 5′-CTAGGAGCCCAGTGGCGA-3′. 

The following PCR conditions were used: 98 °C for 30 s, followed by 35 cycles of 98 °C for 10 s, 63 °C for 20 s and 72 °C for 30 s, followed by a final extension at 72 °C for 10 min.

All PCR products used in the following cloning steps were purified by using the QIA quick gel purification kit (Qiagen), with one exception (see below). PCR fragments corresponding to each gene were first cloned into the pENTR/D-TOPO Gateway recombination vector (Invitrogen), following the standard protocol included in the kit. Using the Gateway LR Clonase Enzyme kit (Invitrogen), each of the *Zeus* or *CAF40* CDS was then recombined in vitro into the pAFW vector from the Bloomington *Drosophila* Genomics Resource center (https://dgrc.bio.indiana.edu/Home, accessed on 1 November 2014), thus placing each CDS in frame with the Actin5C promoter and an N-terminal 3xFLAG tag, as well as the SV40 PolyA sequence at the 3′-end. The combined promoter-tag-CDS-SV40 fragment from each pAFW vector was then amplified via PCR so as to incorporate *PacI* sites at each end of the fragments as follows. Just prior to PCR, the pAFW backbone was digested by using *PmeI* and *SapI* (New England Biolabs). Primers sequences for the PCR are as follows: 

Act5C_PacI forward primer: 5′-ACGTACTTAATTAAGCATGCAATTCTATATTCTAAAAACAC -3′; 

SV_polyA_PacI reverse primer: 5′-ACGTACTTAATTAAGATCCAGACATGATAAGATACATTGAT -3′. 

The following PCR conditions were used: 98 °C for 30 s, followed by 36 cycles of 98 °C for 10 s, 62 °C for 20 s and 72 °C for 1 min and 15 s, followed by a final extension at 72 °C for 10 min. Following PCR, 3′-A overhangs were added to each promoter-tag-CDS-SV40 PCR product, using *Taq* polymerase (Invitrogen). The modified fragments were then isolated by using the S.N.A.P. crystal violet gel purification kit (Invitrogen) and sub-cloned into the PCR-XL-TOPO vector (Invitrogen). The PCR-XL vectors were then digested with *PacI* (New England Biolabs), following standard protocols. Each fragment was then ligated, respectively, into the piggyBac vector MWpBacFPNS (Bloomington *Drosophila* Genomic Resource Center, https://dgrc.bio.indiana.edu/Home, accessed on 1 November 2014) at its *PacI* site. Note that the digested MWpBacFPNS backbone was dephosphorylated (using Antarctic phosphatase, New England Biolabs) prior to ligation, and the ratio (in ng) of backbone to insert in each ligation reaction was approximately 10:1 (excess of piggyBac backbone). After validating the sequence of each completed MWpBacFPNS vector (see below), aliquots of each vector were isolated in a large scale, using the Plasmid Maxi Kit (Qiagen). In addition, a maxiprep scale aliquot of the helper plasmid phsp was also prepared (Handler, personal communication) [19].

At each cloning step, the frame and sequence of the cloned vector were validated via Sanger sequencing, using the following primers: 

ACTf forward primer: 5′-GAGCATTGCGGCTGATAAGG-3′; 

SVr reverse primer: 5′-GGCATTCCACCACTGCTCCC-3′. 

These primer sequences, as well as more information about the Gateway vector system, can be found at: https://emb.carnegiescience.edu/drosophila-gateway-vector-collection, accessed on 1 November 2014). Chromatograms were assembled, aligned and examined by using the software package Geneious v6.0 (available from www.geneious.com, accessed on 1 November 2014). A schematic of the vector construction workflow is presented in Supplementary Figure S1. 

### 2.2. Injections and Screening for Transgenics

All injections were performed by Rainbow Transgenics, Inc. (Camarillo, CA, USA). DNA was injected into embryos at a concentration of approximately 1 mg/mL, with a vector-to-helper (MWpBacFPNS:phsp) ratio of 3:1. Heat-shock induction of the piggyBac transposon was performed at 37 °C, three hours after injection. Strain used for injection was w1118 (*D. melanogaster*). Surviving embryos were reared to adulthood on standard molasses media and backcrossed to their white-eyed parental line. Positive transformants were screened for red eyes (mini-*white* marker) and EGFP in the eyes and ocelli, using an Olympus SZX7 stereomicroscope with mercury lamphouse and reflected fluorescence filters for GFP detection. An example image of a positive transformant is presented in Supplementary Figure S2.

Expression of transgenes was confirmed via reverse transcription, followed by PCR. RNA was isolated, as before, using the Rneasy Mini Kit (Qiagen) with an on-column DnaseI digestion, followed by cDNA synthesis by using the SuperScript III Reverse Transcriptase kit (Invitrogen). For *D. simulans* Zeus expression, RT-PCR was performed by using the following primers and conditions: 

Forward primer: 5′-GATTACAAGGATGACGATGACAAG-3′; 

Reverse primer: 5′-CTAGGAGCCCTCTGTCGACTC-3′; 

Conditions: 95 °C for 2 min, followed by 30 cycles of 95 °C for 30 s, 51 °C for 30 s, 72 

°C for 1 min, followed by 72 °C for 5 min. 

For *D. melanogaster* Zeus expression, primers and conditions were as follows: 

Forward primer: 5′-GATTACAAGGATGACGATGACAAG-3′; 

Reverse primer: 5′-CTAGGAGGAGCCCATTGG-3′;

Conditions: 95 °C for 2 min, followed by 30 cycles of 95 °C for 30 s, 53 °C for 30 s, 72 

°C for 1 min, followed by 72 °C for 5 min. 

For *D. yakuba* CAF40, primers and conditions were: 

Forward primer: 5′-GATTACAAGGATGACGATGACAAG-3′; 

Reverse primer: 5′-CTAGGAGCCCAGTGGCGA-3′;

Conditions: 95 °C for 2 min, followed by 30 cycles of 95 °C for 30 s, 53 °C for 30 s, 72 

°C for 1 min, followed by 72 °C for 5 min. 

An illustrative example of the RT-PCR results is presented in Supplementary Figure S3.

We found that Dmel_Zeus and Dyak_Caf40 protein-coding sequences, as usual, are associated with a very low level of variation compared to the reference sequence. Each contain one nonsynonymous change. Using the DGRP, we confirmed that the Dmel Zeus polymorphism was segregating at high frequency (~50%), suggesting that it was not deleterious. Additional sequencing of *D. yakuba* lines present in the lab suggested that the nonsynonymous polymorphism was present at high frequencies as well.

### 2.3. Chromatin Immunoprecipitation and Sequencing

ChIP-Seq experiments were performed by using standard modEncode protocols after collecting adults in each species. Sequencing data were generated by the High-Throughput Genome Analysis Core (HGAC) at the Institute for Genomics and Systems Biology. All sequencing data are available at GEO, under accession number GSE192880 and GSE192879.

Chromatin isolation followed by immunoprecipitation was carried out by following protocols established by the IGSB at The University of Chicago for modENCODE [20]. Briefly, 600 adult flies were collected of each transgenic genotype (Dsim_Zeus, Dmel_Zeus, and Dyak_Caf40) and divided into 4 sets of 150 (hence, 4 technical replicates per experiment). Crosslinking was performed by homogenizing flies on ice, using both Broeck-type and Dounce-type tissue grinders in Buffer A1 (60 mM KCl, 15 mM NaCl, 15 mM HEPES pH 7.6 4 mM MgCl_2_, 0.5% Triton X-100, 0.5 mM DTT, Roche complete EDTA-free protease inhibitor) with 1.8% formaldehyde. Samples were sonicated (Diagenode sonicator) for 15 min at high power, cycling between on and off every 30 s. Chromatin isolated at this stage was stored at −80 °C. 

Immunoprecipitation (IP) was performed by using 10 mg of rabbit polyclonal anti-FLAG antibody (Sigma) and protein G beads (GE Healthcare) thoroughly washed with lysis buffer (140 mM NaCl, 15 mM HEPES pH 7.6, 1 mM EDTA, 0.5 mM EGTA, 0.1% sodium deoxycholate, 1% Triton X-100 (Sigma-T8787), 0.5 mM DTT, Roche complete EDTA-free protease inhibitor). For each experiment (i.e., each set of 4 replicates for a given factor), an aliquot of chromatin was set aside to which no antibody was added. This input control was sequenced in parallel as a negative IP control. Following the IPs, formaldehyde crosslinks were reversed by heating the samples to either 65 °C (IPs) or to 60 °C (inputs) overnight. DNA isolation was performed first with a phenol:chloroform:isoamyl alcohol (25:24:1) extraction, followed by a chloroform wash, and then precipitated with 100% ethanol and centrifugation at 4 °C. 

Libraries for sequencing were prepared by using the Ovation Ultra-Low Library System (NuGEN) and sequenced on the Illumina HiSeq platform by IGSB at The University of Chicago.

### 2.4. Sequencing and Read Mapping

ChIP-Seq reads were mapped with BWA30, using default parameters, to the most recent UCSC genome versions. Motif discovery was performed with DREME16. We sequenced single-end ChIP libraries on the Illumina GAII platform, with 36 base pair reads. We checked sequencing quality by using FASTQC (“Babraham Bioinformatics—FastQC a Quality Control Tool for High Throughput Sequence Data” 2018). ChIP-Seq reads were mapped with BWA, using default parameters (-n 0.04 -k 2 -M 3 -O 11 -E 4) against the Drosophila melanogaster genome (UCSC dm3) [21].

### 2.5. Signal and Peak Calling

In order to use MEME, we performed peak calling by using MACS2 with the following parameters: -q 0.01 -m 5100. We considered peaks which intersected between the two best replicates, giving us a conservative set of peaks on which to perform motif analysis. We also varied the q-value threshold and found that the CAZAM motif discovery was insensitive to this parameter. 

We called signal on a gene-by-gene basis, using the bedtools [22] coverageBed command. For each gene with a transcription start site annotated in the Eukaryotic Promoter Database [23], we extended its TSS 350 base pairs in each direction. We then counted the number of mapped reads falling within each of these regions. To eliminate the effect of different sequence depths in different experiments, we scaled each promoter’s signal to the mean signal in that experiment. Reported results were consistent when we varied the width of the window around each TSS (we examined 100, 500, and 1000 bp extensions.

We saw the same results when analyzing read depth within exons of annotated genes, as well as using only called peaks.

### 2.6. Sex-Bias and Spermatogenesis Expression

We used the SEBIDA database to determine individual gene’s sex-bias pattern [24]. Genes were called male-biased, female-biased, unbiased or unclassified according to the meta q-value of the previous study [24]. 

We employed the SpPress database of the previous study to determine Zeus’s effect on gene expression in the developing testis (Supplementary Figure S4) [25].

To analyze the relative sex-bias of *D. melanogaster* and *D. simulans*, we used microarray data from Ranz et al. (2003) [26]. Using the ratio of male-to-female expression, we found that the 4th chromosome contained genes that were, on average, significantly more female-biased in melanogaster than in *D. simulans* (permutation test, *p* < 0.01).

### 2.7. DREME

To detect motifs, we used the software DREME. We collected sequence from each of the three proteins’ peaks and ran DREME on these sequences separately. We used the following parameters: sites of width >6 and <50, expecting zero or one occurrence per sequence. Full motifs can be found in Supplementary Figures S5–S7. Because we determined that sites flanking the core motif (see “2.8. Motif Analysis”) demonstrated variable base pair preference and information content depending on the peak set and threshold used, we considered only the core, which was invariant (‘ACTGCTT’), for further analyses. 

Additional motifs were found for each of the three proteins, some of which may contribute to the varying binding preferences we discovered in the ChIP-Seq data. However, we chose to focus on the core conserved site that was held in common between all three proteins.

### 2.8. Motif Analysis

Using DREME, we found strong enrichment for a seven-base pair core motif, ACTGCTT, which we term the CAZAM (for *Caf40* and *Zeus*-Associated Motif). To further examine the distribution of the CAZAM, we used custom Perl scripts to look for all occurrences of this motif (as well as its reverse complement) in the genomes of *D. melanogaster* and its closest relatives. We used nine total genomes from UCSC, all the most recent releases (Supplementary Table S2). 

To test for differences in motif abundance between genomes, we used the phylogenetic ANOVA [27], as implemented in the R package phytools [28]. The phylogenetic ANOVA accounts for relatedness between species and tests the hypothesis that the motif evolved under a simple one-rate Brownian motion model of evolution. The Brownian motion model is a kind of “random walk” model. The trait values on this model vary randomly in both direction and distance over time interval. The key part of biological models of evolution in terms of Brownian motion is that the motion of the object is due to the sum of a large number of weak random forces. Based on phylogenetic ANOVA analysis, a significant result indicates a pattern of evolution which is inconsistent with Brownian motion model. However, if not significant, it does not mean not evolve, but evolve with a pattern does not like “random walk” by potential main forces. 

We did the same analysis of motif frequency, but using the most recent FlyBase versions of each genome. We obtained qualitatively similar results by using these genome versions instead of the UCSC genome equivalents (Supplementary Figure S8).

### 2.9. Exon Bias of Motifs

Post- and pre-duplication species (in particular, *D. simulans* and *D. yakuba*) showed a marked difference in the frequency of CAZAMs within 1 kb of exons (using UCSC xenoRefGene annotation). To correct for annotation differences between species (*D. simulans* had more than double the total amount of annotated sequence), we randomly downsampled *D. simulans* annotations, so that they covered the same total amount of annotated sequence as *D. yakuba* annotations. Even after downsampling in this manner, *D. simulans* showed a significantly greater proportion of CAZAM motifs were within 1 kb of exons. We note that the *p*-value (derived from using Fisher’s Exact Test) in this case is approximate because of the random nature of the downsampling procedure; to be conservative, the reported *p*-value is the highest *p*-value observed from a set of 10 iterated downsamplings.

### 2.10. Promoter Motif Frequency Analysis 

We analyzed the promoters of *D. melanogaster* genes that contained at least one peak in our conservative peak set, which we define as Zeus-bound genes (see the section of Signal and Peak Calling in Materials and Methods). We produced bed files for the promoter regions of *D. melanogaster* Zeus-bound genes and their respective orthologs in *Drosophila wilistoni*, *Drosophila pseudoobscura*, *Drosophila ananassae*, *Drosophila. virilis*, *D. yakuba* and *D. simulans.* Promoter regions were defined as being 500 bp upstream of the TSS for this analysis and were created by using bedtools v2.29.1 [29], samtools 1.13 [30] and gff2bed 2.4.40 [31]. We counted the number of CAZAM motif instances in promoters of *D. melanogaster* Zeus-bound genes and their orthologs in six Drosophilids with FIMO 5.0.5 [32].

### 2.11. Statistical Analysis

To test for differences in mean or median between groups, we used permutation tests, also called “sampled randomization tests” [33] (p. 808). All statistical analyses were conducted using the R programming language.

### 2.12. Population Genetics

We used SNP calls from the DGRP23, filtering variants with minor allele frequency less than 0.05 to remove weakly deleterious variation. We located all motif instances in genomes of different species, using a custom Perl script. Using bedtools slopBed (-d 7), we extended each match seven base pairs in each direction, producing segments 21 base pairs in length. We then mapped motif instances between genomes by using the software liftOver, with the minMatch parameter set at the default value of 0.95. 

Our analysis discarded motif instances located in heterochromatic segments or on unassembled contigs (“chrU”), reasoning that alignment would be unreliable for motifs located in these regions. We retrieved orthologous sequence on the main chromosome arms from the other species and calculated the number of fixed differences. We considered alignments of each sequence in both the forward and reverse orientation, selecting the alignment which minimized the total number of differences.

To determine the number of polymorphic sites, we utilized data from the Drosophila Genetic Reference Panel [34]. For results reported in the paper, we considered only single nucleotide polymorphisms of minor allele frequency greater than 5%, omitting indel mutations. Results were similar when we incorporated indels and varied the minor allele frequency thresholds (see Supplementary Tables S3–S5). 

Our version of the McDonald–Kreitman test uses the motif as dN and the flanking regions on either side as dS. It is important to note that, by using immediately flanking sequences, our version of the McDonald–Kreitman test may be biased against detecting selection. In the canonical form of the test [35], dS (analogous to dF) is used to refer to synonymous sites which are under weak [36] or no selection. In contrast, in our formulation of the test, the sites immediately adjacent to each motif may be bound by other trans-acting factors, or code for proteins. On average, these flanking sites show a SNP density and minor allele frequency much lower than that of synonymous sites, potentially indicating non-neutral regimes of evolution. When we instead used nearby synonymous sites as a neutral reference [9], we found significantly higher estimates of α (Supplementary Table S6).

In all cases, reported confidence intervals for α are based on bootstrapping [37], that is, repeatedly and randomly sampling the sequences with replacement. We also used G tests and Fisher’s Exact Test to assess the significance of polymorphism and divergence, following McDonald and Kreitman, 1990 [35]; in each case, results were similar. 

As a further means of confirming that the CAZAM was under uniquely strong selection, we performed the same tests on all two base-pair shufflings of the CAZAM (i.e., ACTGTCTT -> ACGTCTT, CATGCTT, etc.). We ran these shufflings through the same pipeline as described above, noting the resulting value of α (see Supplementary Table S7). No randomly generated motif showed mean α values as high as the CAZAM, and no motif showed a significant test result for both *D. yakuba* and *D. pseudoobscura*, suggesting that the CAZAM was under uniquely strong selection following the origination of *Zeus*.

### 2.13. CRISPR-Cas9 Mediated Zeus Deletion

We created CRISPR indel mutation within the CDS sequence of Zeus by following the methods outlined in these two studies [38,39]. Briefly, two guide RNAs primers (gRNAs, gs17F and gs18F) were designed by using the FlyCRISPR Optimal Target Finder [40]. We amplified the gDNAs by combining the universal reverse primer sgRNA_R (Phusion™ High-Fidelity DNA Polymerase (2 U/µL), Catalog number: F-530XL) and synthesized the gRNAs using the Invitrogen™ MEGAshortscript™ T7 Transcription Kit (Catalog No. AM1354). Using a microinjector, we microinjected the two gRNAs (~300 ng/μL each) together with Cas9 protein (500 ng/μL, PNA BIO INC, #CP01) into preblastodermDrosophila melanogaster embryos (BDSC #25710; P{y[+t7.7] = nos-phiC31\int.NLS}X, y[1] sc[1] v[1] sev[21]; P{y[+t7.7] = CaryP}attP2). High-Resolution Melting Analysis (HRMA) was used to screen the potential T0 positive mutants. Small frameshift deletions were confirmed through Sanger sequencing and created early stop codons in the transcribed genes.

gs15F:5’-GAAATTAATACGACTCACTATAGGCTGCTGGGGACTCATTACGTTTAAGAGCTATGCTGGAA-3’;

sgRNA_R:5’-AAAAGCACCGACTCGGTGCCACTTTTTCAAGTTGATAACGGACTAGCCTTATTTAAACTTGCTATGCTGTTTCCAGCATAGCTCTTAAAC-3’; 

hrma_F: CCAAGCATCCATCTGTTTAATGGG

hrma_R: CAGGATAGGCCAGCTCGATG

### 2.14. RNA Extraction, Library Preparation and Differential Expression Genes Analysis

We extracted RNA from whole testes from our Zeus CRISPR deletion lines and control injection line in biological triplicate, using the ArcturusTM PicoPureTM RNA Isolation kit (Applied Biosystems, LOT 00665884). Then 1 μg of RNA per each of the six samples was used to construct the cDNA library by using NEBNext Ultra RNA Library Prep Kit for Illumina (NEB, #E7770), following manufacturer’s introductions. Briefly, poly(A) mRNA was purified from total RNA, using oligo(dT)-attached magnetic beads; reverse-transcribed to double-stranded cDNA with random primers; end-repaired; and ligated with NEB adaptors for Illumina sequencing (HiSeq 4000, University of Chicago Genomics Core Facility).

The quality of raw sequencing data was assessed by using FastQC (“Babraham Bioinformatics—FastQC a Quality Control Tool for High Throughput Sequence Data” 2018). Overall, QC reports of all data generated by FastQC indicate high confidence of sequencing results on the call (Supplementary Table S8). Illumina adapters/primers sequences were detected from sequencing reads. All RNA reads were first mapped to *D. melanogaster* reference genome (dm6) by using STAR with default parameters [41]. Picard was used to collect mapping metrics. The evaluation of transcriptional expression was carried out by using featuresCounts [42]. Several state-of-the-art tools, including DESeq2 [43], edgeR [44] and limma [45], were independently employed for the differential expression genes (DEGs) analysis. We defined genes as being “differentially expressed” if they were consensually called by the three methods, with an expression fold change of at least 1.5 compared to the control at false discovery rate less than 0.05 in knockout samples compared to control samples (Supplementary Table S9). 

For DEGs, enriched biological processes and molecular functions were identified by using PANTHER Overrepresentation Test [46], with *p*-values < 10^−4^, and a false discovery rate of 0.05.

## 3. Results

### 3.1. Divergent ChIP-Seq Profile between D. melanogaster Zeus, D. simulans Zeus and D. yakuba Caf40

Our previous work has shown that Zeus acquired a significant number of species-specific substitutions in *D. melanogaster* and *D. simulans*. Thus, we hypothesized that these changes may result in different DNA binding profiles. 

To compare the binding properties of *D. melanogaster* Zeus, *D. simulans* Zeus and *D. yakuba* Caf40, we engineered transgenic lines of *D. melanogaster* (w1118) that contain FLAG-tagged *D. melanogaster Zeus*, *D. simulans Zeus* and *D. yakuba Caf40* and performed ChIP-Seq on each of these lines (Figure 1). This allowed us to directly compare binding properties of the three proteins in a common genome. We term these three proteins Dmel_Zeus, Dsim Zeus and Dyak_Caf40, respectively. We obtained reproducible ChIP-Seq signals between replicates (Supplementary Figure S10 and Table S1). 

**Figure 2:**
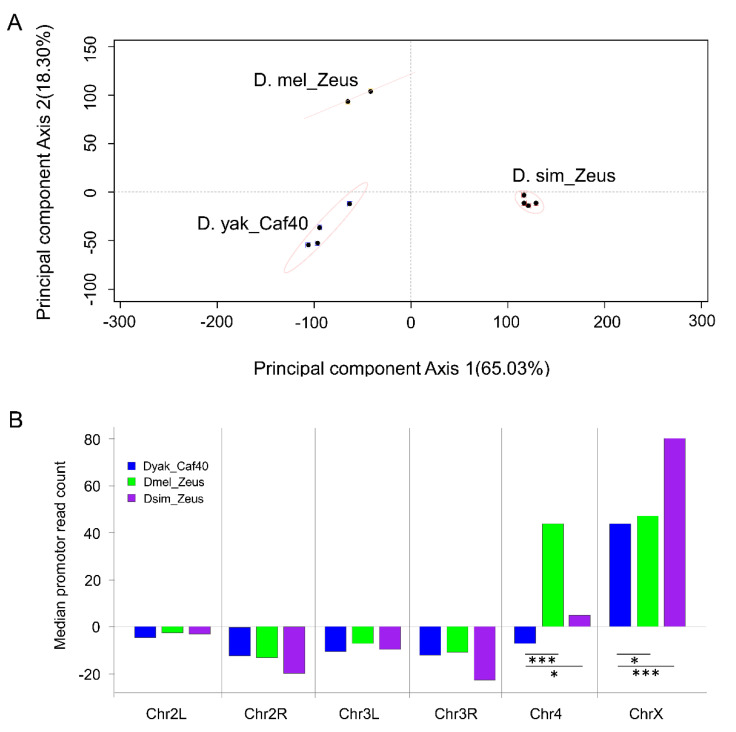
Evolution of *Zeus* binding affinity in trans. (**A**) Graph of the first two principal components of ChIP-Seq read counts revealed reproducible clustering of replicates of the same protein, while different proteins showed differentiation. (**B**) Bar plot showing the median normalized read counts over TSSs for each chromosome, indicating differences in chromosome-level affinity of the three proteins. Both copies of *Zeus* show increased affinity relative to *Caf40* on chromosomes X and 4, albeit to different degrees. *t*-test: * *p* < 0.05, *** *p* < 0.001. Error bars indicate SD.

We observed a higher degree of correlation between testis gene expression and observed Zeus binding compared to *D. yakuba* Caf40 (Supplementary Figure S2). We observed strong enrichment of ChIP signal primarily at the transcription start site and within the exons of bound genes (Supplementary Figure S11), refining previously hypothesized Zeus and Caf40 binding preferences [16]. To assess the potential differences in binding among the three proteins, we calculated signal enrichment for each gene based on the enrichment of reads within 700 bp of the transcription start site (TSS). Principal component analysis on the gene-by-gene signal revealed that replicates corresponding to each protein (Dmel_Zeus, Dsim_Zeus and Dyak_Caf40) formed distinct clusters (Figure 2A), demonstrating significant differences in binding preferences between proteins. We computed the pairwise Euclidean distance between proteins’ read counts, which showed that Dsim_Zeus sites were more highly diverged from Dyak_Caf40 than Dmel_Zeus sites (Supplementary Figure S12; *p* < 0.001), as is consistent with the reported pattern of protein-coding sequence divergence (Figure 1).

### 3.2. Zeus Gained Affinity for Sex-Biased Genes on Both X Chromosome and Chromosome 4

Based on previous ChIP-Seq results showing that Zeus preferentially binds the X chromosome, and because of the known roles of Zeus in regulating sex-specific functions (which are enriched on the X chromosome), we compared the chromosomal distribution of reads [47]. We found ChIP-Seq read enrichment for all three proteins on the X chromosome relative to the autosomes (Figure 2B), but both Zeus orthologs showed significantly higher X vs. autosome signal enrichment compared to Dyak_Caf40 (permutation test: *p* < 0.05). Dsim Zeus showed particularly strong X chromosome enrichment (permutation test: *p* < 0.001).

Both Zeus proteins also exhibited a bias for the fourth (dot) chromosome—which has been hypothesized to be an ancestral sex chromosome [48,49]—while Dyak_Caf40 does not. The pattern of bias mirrored that observed for the X chromosome: Dmel_Zeus was strongly enriched for signal on the fourth chromosome (*p* < 0.001), whereas Dsim Zeus was mildly, but significantly, enriched (*p* < 0.05). Both the X and dot chromosomes are enriched for female-biased genes [24] (Fisher’s Exact Test: *p* = 2.728 × 10^−14^), as is consistent with *Zeus*’s hypothesized repressive role in the testes [50]. The chromosomal distribution of sites thus suggests a scenario in which *Zeus* gained an affinity for sex-biased genes on the X chromosome and the fourth chromosome as part of its testis-specific neofunctionalization and then subsequently evolved differences in chromosome level binding between *D. melanogaster* and *D. simulans*. Genes on *D. melanogaster*’s fourth chromosome were found to be, on average, more highly female-biased than *D. simulans*, explaining the significant species-specific difference in affinity [26] (see Supplementary Materials; permutation test, *p* < 0.05).

### 3.3. Zeus-Derived Genome-Wide Frequency of CAZAM-Motif Variation and Motif Redistribution between Drosophila Species with and without Zeus Gene

Caf40 is among the most conserved nucleic acid–binding proteins across eukaryotes, from metazoans to fungi to flowering plants [51]. We reasoned that the extensive protein-coding (trans-) divergence of *Caf40* and *Zeus* may have driven the evolution of conserved bound cis-regulatory elements [52]. We therefore searched for overrepresented motifs for each protein, using DREME [53]. A single highly specific motif (ACTGCTT) was enriched in all three proteins’ binding sites (Supplementary Figures S5–S7). We call this motif the Caf40 and Zeus-Associated Motif (CAZAM).

**Figure 3:**
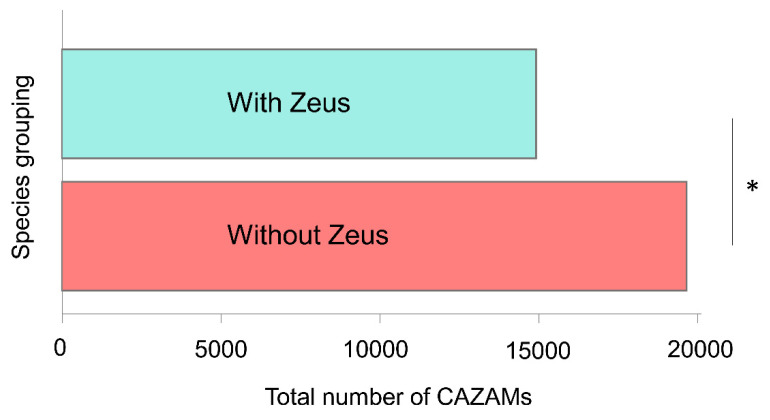
CAZAM frequency varies between species that possess and do not possess *Zeus*. Bar plot showing the mean frequency of the CAZAM in species with (top bar) and without (bottom bar) the *Zeus* duplication. The frequency of the CAZAM is significantly lower in species with *Zeus* (* *p* < 0.01).

We noted that the genome-wide frequency of the CAZAM differed between *Drosophila* species with and without the *Zeus* gene. The three species of sequenced *Drosophilids* with both the *Zeus* and *Caf40* genes had significantly lower overall CAZAM frequencies than sequenced species with only *Caf40*, which remained true after correcting for genome size (Figure 3; Supplementary Table S2; phylogenetic ANOVA, *p* = 0.004). No randomly constructed motifs were similarly unevenly distributed among the genomes (Supplementary Figure S13).

While the genome-wide frequency of the CAZAM was lower in *Drosophila* species that contained the *Zeus* gene as compared to those without the *Zeus* gene, we found that the frequency of motifs in the promoters (defined as 500 bp from the beginning of the transcription start site) of all *D. melanogaster* Zeus-bound orthologs was highest in *D. melanogaster* and *D. simulans* and lowest in orthologous promoters of *Drosophilids* that did not contain Zeus (Supplementary Figure S9). 

In addition to an overall difference in CAZAM frequency, we found that the distribution of the motif was radically different among the genomes with and without *Zeus*. After the origination of *Zeus*, the frequency of CAZAMs on the X chromosome did not change appreciably, while motifs decreased on the autosomes (Supplementary Table S2). The fraction of motifs within 1 kb of exons, on both X and autosomes, increased dramatically as well (97.8% in *D. simulans* vs. 84.0% in *D. yakuba*; Fisher’s Exact Test: *p* < 1 × 10^−7^). The increase in exon proximal binding may be indicative of a refining of target specificity to those genes required for *Zeus*’s new function. These results suggest that selection acted to impose on reorganization of thousands of copies of the motif following the gene duplication event, perhaps because of a new regime of selection driven by the emergence of *Zeus*.

### 3.4. Origination of Zeus Reshaped Selection Pressure Variance of the Motif across Species

**Figure 4:**
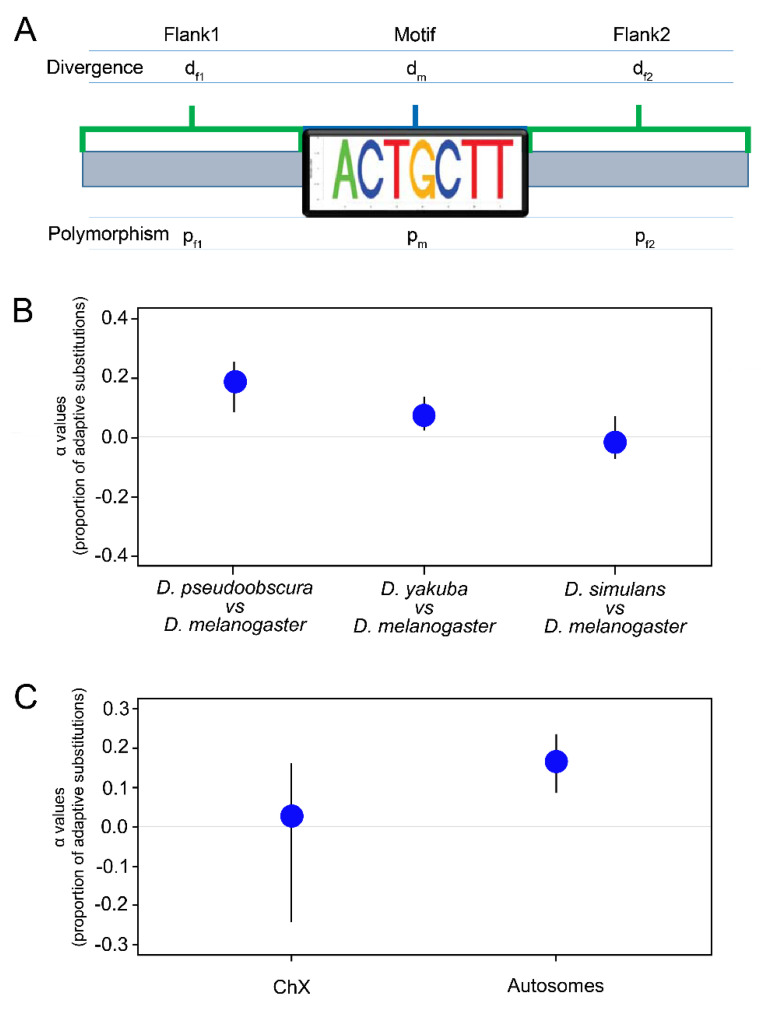
CAZAM exhibits a signature of selection. (**A**) Schematic illustrating our modification of the McDonald–Kreitman test. We substitute central and flanking sites for dN and dS, respectively, allowing us to measure selection on all identified instances of the motif. (**B**) Plot depicting observed α (α) values (interpreted as the proportion of adaptive substitutions) for different comparisons, with bootstrapped 99% confidence intervals. Motifs located and mapped from *D. pseudoobscura* and *D. yakuba* show values of α significantly different from zero, while motifs from *D. simulans* do not. (**C**) Comparison of estimated α values from motifs which map to the X chromosome (left) in contrast to those which map to the autosomes (right). Values of α are significantly lower—indicative of weaker selection—for motifs located on the X chromosome.

To assay for positive selection more directly, we modified the framework of the McDonald–Kreitman test so that it could apply to motif-level analyses at a whole-genome scale [54,55,56,57] (Figure 4A; see Supplementary Materials). Because we posited, based on the overall difference in motif frequency between species, that there was selection to impose on reduction of the motif from the genomes of species after *Zeus* duplicated from *Caf40*, we identified motif instances in pre-duplication species (*D. yakuba*, *D. pseudoobscura*), as well as one post-duplication species (*D. simulans*), and mapped their syntenic locations into *D. melanogaster*. We note that our version of the test may be conservative, as the flanking regions surrounding motifs showed evidence of stronger purifying selection than synonymous sites, the usual reference for the McDonald–Kreitman test [36].

Using divergence data from whole-genome multiple alignments between each compared species and *D. melanogaster*, and polymorphism data for *D. melanogaster* [34], we found that there was significant evidence of positive selection on instances of the CAZAM following the gene duplication event (Figure 4B; *D. pseudoobscura*–*D. melanogaster*, bootstrap test, *p* < 0.01; *D. yakuba*–*D. melanogaster*, bootstrap test, *p* < 0.01). Selection was significantly stronger on intergenic motifs than on exonic motifs, as is consistent with our findings that all three proteins were bound near exonic regions and that there was redistribution of the motif following the duplication event (Supplementary Table S3; *p* < 0.01). In contrast, performing the same comparison between *D. simulans* and *D. melanogaster* revealed no significant signature of positive selection, suggesting that strong selection acted after the duplication event, but decreased by the time the *D. melanogaster* and *D. simulans* lineages diverged [2,58] (Figure 4B).

Because we determined earlier that Zeus binding shows a strong chromosome-specific bias consonant with its role in testes development [59], we posited that regimes of selection may have differed across chromosomes. Correspondingly, we found evidence of stronger selection to impose on reduction of CAZAMs from autosomal chromosomes than from the X chromosome (Figure 4C; comparing intergenic motifs in *D. yakuba* and *D. melanogaster*; permutation test, *p* < 0.01). We conclude, based on the motif frequency difference and associated evidence of positive selection, that widespread selection driven by the origination of *Zeus* shaped both the abundance and distribution of the motif between species.

To confirm that this signature of selection on the CAZAM was related specifically to the appearance of *Zeus*, we performed a series of control analyses. We examined several motifs that were shuffled versions of the CAZAM and found significant McDonald–Kreitman test results for none of them after multiple testing correction (bootstrap tests, *p* > 0.5; see Supplemental Materials, specifically Supplementary Tables S4–S7). We examined sequence divergence of the CAZAM between two species (*D. pseudoobscura* and *D. yakuba*) without *Zeus* and found no significant signal of increased divergence at motif sites relative to flanks (Supplementary Table S7; Bootstrap test: *p* > 0.5). Our results therefore suggest that it was the origination of *Zeus* that led to widespread positive selection specifically on the CAZAM.

### 3.5. Zeus-Regulated Gene Expression Does Not Depend on CAZAM Binding in the Whole Testes

**Figure 5:**
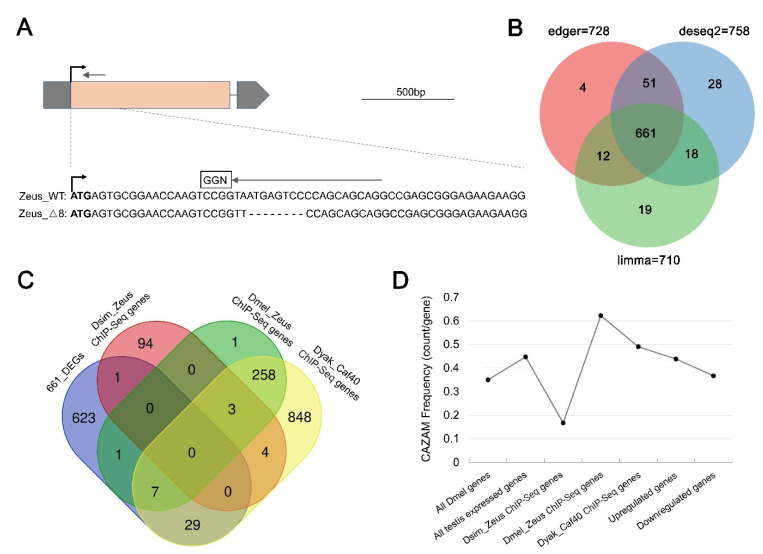
Zeus regulated DEGs expression not by direct CAZAM Motif binding pattern. (**A**) Zeus_KO CRISPR mutant creation. Small gray arrow indicates position of small-guide RNA. Peach section corresponds to the one and only exon. Gray sections correspond to the 5′ and 3′ UTR. (**B**) Venn Diagram of DEGs identified in three differential expression analysis software: EdgeR, DEseq2 and Limma (see Supplementary Materials and Methods). (**C**) Venn Diagram among the 661 DEGs, 102 Dsim_Zeus ChIP-Seq genes, 270 Dmel_Zeus ChIP-Seq genes, and 1149 Dyak_Caf40 ChIP-Seq genes. (**D**) CAZAM frequency in several gene groups: all Dmel genes refer to all annotated 17,874 *D. melanogaster* genes; all testis expressed genes refers to testes expressed 11,491 genes by RNA-Seq in this study; 331 upregulated genes and 330 downregulated genes constitute the 661 DEGs in Zeus_KO testes by RNA-Seq in this study.

Given the extensive positive selection on the CAZAM following the origination of Zeus, we hypothesized that Zeus directly binds CAZAM to regulate gene expression in the testes. To test this, we generated *Zeus* loss-of-function lines by using CRISPR-Cas9 (see Methods section and Figure 5A). We found that the KO lines had significantly reduced (22%) viability (*p* < 0.05, t-test) [13], suggesting an important functional role of Zeus. We further conducted RNA-Seq on adult male whole testes, in which *Zeus* is normally expressed, from control injection and *Zeus* knockout (KO) lines in biological triplicate. 

We identified 661 differentially expressed genes (DEGs) between *Zeus* KO and control testes (Figure 5B, Supplemental Table S9). In total, 331 DEGs were upregulated, while 330 DEGs were downregulated in KO samples compared to our controls. Gene ontology analysis revealed that downregulated DEGs were enriched for cellular metabolic processes and gene expression (Supplementary Table S10). Notably, we observed 38 genes in total overlapping with the genes bound by the three sets of ChIP-Seq of Dmel_Zeus, Dsim_Zeus and Dyak_Caf40 (Figure 5C), especially 36 out of 38 genes overlapping with Dyak_Caf40. However, we did not observe a great degree of overlap between the 661 DEGs in *Zeus* KO samples and the three sets of ChIP-Seq binding genes, suggesting that *Zeus* regulation of a large majority of the 661 DEGs is not directly through CAZAM-binding (Figure 5C). We calculated the frequency of CAZAM occurrence in seven gene groups: all annotated *D. melanogaster* genes (total 17,874 genes), all testis expressed genes (total 11,491 genes), 102 Dsim_Zeus ChIP-Seq genes, 270 Dmel_Zeus ChIP-Seq genes, 1149 Dyak_Caf40 ChIP-Seq genes, 331 upregulated genes and 330 downregulated genes (Figure 5D). Both upregulated and downregulated DEGs did not show a significant enrichment of CAZAM relative to all other genes expressed in the testes (Figure 5D). These results suggest that Zeus binding of CAZAM is not necessary for gene regulation in the whole testes, but we cannot exclude that it may be necessary for the regulation for specific cell types in the testes (Supplementary Figure S2 from Vibranovski et al., 2009b) [60]. We also observed that the numbers of both Dmel_Zeus ChIP-Seq genes and Dsim_Zeus ChIP-Seq genes are much lower than Dyak_Caf40 ChIP-Seq genes (Figure 5C). The CAZAM frequency of both Dmel_Zeus ChIP-Seq genes and Dsim_Zeus ChIP-Seq genes shows a drastic fluctuation (Figure 5D). Taken together, we speculate that Zeus exhibits a rapid, dynamic and species-differential coevolution with specific motif for its neofunctionalization, as is consistent with what we recently observed [13].

## 4. Discussion

Our results show that Zeus, a novel nucleic acid–binding factor in *Drosophila*, underwent a regime of rapid neofunctionalization, ultimately leading to specialized binding to different chromosomes in different species. This trans-evolution, in turn, drove strong positive selection to rearrange the chromosomal distribution of the motif associated with both Zeus and Caf40 binding. We have thus revealed a dynamic genome-wide coevolutionary process of neofunctionalization occurring in both *cis* and *trans*.

With regard to the specific molecular mechanism by which *Zeus* might be regulating downstream targets, initial studies of *Caf40* (also known as *Rcd-1*) suggested that it regulates target genes through direct interaction with the genome, due to the fact that it contains six armadillo-type repeats, as implicated in DNA binding [18,61]. Our data show that, via ChIP, we can indeed discover signals of *Caf40* and *Zeus* binding that illuminate their evolutionary histories, although we cannot discount the possibility that the signals we detect could in fact be due to indirect interactions with the genome mediated through protein–protein binding. For example, extensive studies of transcription factors (TFs) binding have revealed that interactions between TFs and the genome are mediated through a highly complex and variable suite of direct and indirect interactions between TFs and cofactors [8,62,63,64,65]. However, several studies suggest that *Caf40* in *Drosophila* may also act indirectly with nucleic acids as a member of the larger CCR4–NOT complex, which has roles in mRNA processing and degradation [66,67,68,69,70]. Dramatically, a recent study conducting a co-immunoprecipitation in Dm S2 cells by expressing a GFP-tagged version of three paralogs (*Caf40* and its two retroduplicates, *Zeus* and *Poseidon*) assayed their interaction with HA-tagged NOT1, which is the central scaffold subunit of the CCR4–NOT complex [13]. Their result suggested that Poseidon conserved Caf40’s ability to interact with the CCR4–NOT complex, while Zeus almost lost its CCR4–NOT recruitment ability [13]. Moreover, mRNA-tethering assay also displayed similar pattern: Poseidon has conserved the same repressive effect on targeted mRNAs observed for CAF40, while Zeus exhibits a significantly weaker repressive ability [13]. 

In this work, we showed that Zeus is required for the expression of CAZAM-enriched genes in the testes, suggesting that Zeus–CAZAM binding is important for regulating gene expression. Therefore, *Zeus* has undergone rapid evolutionary changes both in terms of its protein–protein and protein–nucleic acid interactions. Taking these results together, we see that *Zeus* rapidly underwent both neofunctionalization ( recruiting characteristic cis–trans coevolution) and subfunctionalization ( losing interaction with ancestral conserved CCR4–NOT complex but keeping a decreased mRNA tether ability) in a short evolutionary time of less than 5 million years. We also noted from our analysis that both Dmel_Zeus and Dsim_Zeus show enriched binding on the X and fourth chromosomes, consistent with the putative role of Zeus in the downregulation of female-biased genes. This finding is consonant with the fact that these two chromosomes are heavily heterochromatinized and that Zeus may also be involved in chromatin dynamics [16,17]. The detected difference of binding genes with Zeus and Caf40 by using ChIP-Seq and those DEGs by *Zeus* CRISPR knockout mutant, while likely reflecting a different degree of interaction with the CAZAM, may also hypothetically be a consequence of possible competition between the transgenic genes and wild-type genes in the transformed lines. To test such an impact of competition, it might be illuminating to directly insert both versions of Caf40 and Zeus with different tags for a direct comparison of relative binding.

Regarding the evolution of the CAZAM, one might suggest that there are several important caveats that apply to our version of the McDonald–Kreitman test. Because of the genome-wide nature of our test, we examined many motifs which are likely not bound by either Caf40 or Zeus, due to occlusion by chromatin or other DNA-bound factors. In addition, extending the McDonald–Kreitman test to a genome-wide scale aggregated many unlinked motifs that can have adverse and unpredictable effects upon the bias of the test [71]. However, by creating an empirical null distribution of sequences resembling, but different from, the CAZAM, many of the potential issues with the modified McDonald–Kreitman test can be reduced. If the test was overly liberal in detecting selection, we would expect to see selection on the permuted CAZAM sequences, as well as in pairs of species which did not differ in terms of the presence of *Zeus*. Instead, we find that the null hypothesis is rejected only for the specific motif we found in our ChIP-Seq data, and only in the particular case in which one compares two species across a specific phylogenetic node that corresponds to the origination of *Zeus*.

Our results shed light on the fate of newly arisen functional gene duplicates. From our studies of *Zeus*, we have demonstrated that novel regulatory proteins may cause positive selection to drive genome-scale rewiring of the transcriptional networks into which they integrate through changes both in the protein itself and the global cis-regulatory environment. Overall, these global changes, in turn, can have important phenotypic consequences (e.g., the development and function of the reproductive system), even over short evolutionary timescales. 

## 5. Conclusions

Our results shed light on the fate of newly arisen functional gene duplicates. From our studies of *Zeus*, we have demonstrated that novel regulatory proteins may cause positive selection to drive genome-scale rewiring of the transcriptional networks into which they integrate through changes both in the protein itself and the global cis-regulatory environment. Overall, these global changes, in turn, can have important phenotypic consequences (e.g., the development and function of the reproductive system), even over short evolutionary timescales. 

## Data Availability

Not applicable.

## Supplementary Materials

The following are available online at https://www.mdpi.com/article/10.3390/genes13010057/s1. Figure S1: Vector construction. For each gene, four sub-cloning steps were required to build piggyBac vectors used to engineer transgenic flies. (A) Coding sequence (CDS) of interest (blue) was first cloned into pENTR D-TOPO vector (Invitrogen) following manufacturer’s protocol. (B) CDS was then recombined by using Gateway recombination (Invitrogen) into pAFW vector, resulting in a construct with the CDS in frame with Act5C promoter and the 3xFLAG tag. (C) Actin5c-FLAG-CDS fragment was then TOPO cloned into PCR-XL vector (Invitrogen). (D) Finally, promoter-FLAG-CDS fragment was ligated into MWpBacFPNS vector used for transgenesis. Notes: In (A)–(D) the CDS depicted is Dsim_Zeus as an example; an identical workflow was followed for all other genes. Vector maps are not to scale with each other; size of each vector in base pairs (bp) is noted in the figure. Vector map images were created by using Geneious software (Geneious version 6.0 created by Biomatters. Available from www.geneious.com, accessed on 1 November 2014). Figure S2: Detection of transgenic flies. Top panel: Example of transgenic *D. melanogaster* expressing mini-white marker(right) next to non-trangenic sibling under white light. Bottom panel: Same two flies as top panel photographed under fluorescent light to stimulate GFP. Black scale bar between panels = 0.5 mm. Figure S3: RT-PCRs of transgenic lines. Example of positive RT-PCR results to confirm expression of transgenic inserts. Key: RT+ denotes addition of reverse transcriptase. RT denotes lack of reverse transcriptase (control for DNA contamination). Lane 1: 1 kb ladder (Invitrogen). Lane 2: 2-log ladder (New England Biolabs). Lanes 3–7: Transgenic Dmel_Zeus vector in Dmel, RT+. Lane 8: Transgenic Dmel_Zeus vector in Dsim, RT+. Lane 9: Dmel w1118, RT+ (negative expression control). Lane 10: Dsim w501, RT+ (negative expression control). Lanes 11–15: Transgenic Dmel_Zeus vector in Dmel, RT- (negative DNA carryover controls). Lane 16: Transgenic Dmel_Zeus vector in Dsim, RT- (negative DNA carry-over control). Lane 17: Dmel w1118, RT- (negative control). Lane 18: Water (negative PCR control). Lane 20: 2-log ladder (New England Biolabs). Figure S4: Gene-expression correlations in the testis. Using data from SpPress (Vibranovski et al. 2009b) [32], we examined the correlation between Zeus’s binding in each gene and its expression dynamics in the testis. Zeus expression is known to decline from pre-meiotic to post-meiotic stages, so we hypothesized that genes to which Zeus was strongly bound would show greater ratios of post-meiotic/pre-meiotic gene expression. Accordingly, we discovered significant positive correlations for each protein’s TSS signal and the ratio of post-meiotic/pre-meiotic gene expression, indicating that our ChIP-Seq was capturing information about Zeus’s regulation. Notably, correlations were significantly higher for both versions of Zeus than for Caf40, according well with Zeus’s testis-specific functionality. Bars represent 95% bootstrap confidence intervals. Figure S5: CAZAM motif as determined from Dmel_Zeus data. Logo showing the CAZAM motif, as determined from Dmel_Zeus data, using DREME (see Supplementary Methods for details). Figure S6: CAZAM motif as determined from Dsim_Zeus data. Logo showing the CAZAM motif, as determined from Dsim_Zeus data, using DREME (see Supplementary Methods for details). Figure S7: CAZAM motif as determined from Dyak_Caf40 data. Logo showing the CAZAM motif, as determined from Dmel_Zeus data, using DREME (see Supplementary Methods for details). Figure S8: Motif frequency differences, using different genome versions. As in Figure 2A, but using the most current FlyBase versions of each species’ genomes instead of UCSC genome versions. Motif frequency differences remain significant by phylogenetic ANOVA (*p* = 0.041). Figure S9: CAZAM frequency in the promoters of *Drosophila melanogaster* Zeus-bound genes and their orthologs in *Drosophilids*. Promoters were defined as 500 bp upstream of the transcription start site. Asterisks (*) denote the species that contain *Zeus*. Dmel = *Drosophila melanogaster*, Dsim = *Drosophila simulans*, Dyak = *Drosophila yakuba*, Dana = *Drosophila ananassae*, Dpse = *Drosophila pseudoobscura*, Dwil = *Drosophila willistoni*, Dvir = *Drosophila virilis*. Figure S10: ChIP-Seq data show high between-replicate correlation. Each dot is a single promoter, with read counts in one replicate of a *D. yakuba* Caf40 ChIP-Seq experiment plotted against the read counts in a different replicate of the same experiment. Correlations between replicates were very high (r > 0.95) for all three proteins. Figure S11: ChIP-Seq signal is concentrated at the transcriptional start site of genes. The scaled read-depth normalized mean signal is shown for each protein’s average ChIP-Seq binding pattern in the vicinity of the TSS. The central line represents the transcription start site. Signal is highest at this point, falling off rapidly upstream of the TSS, and remaining strong into the exons of genes (downstream). The three proteins showed no detectable difference in signal as a function of distance from the TSS. Figure S12: Comparisons of Euclidean distance between proteins’ binding affinities. Using the depth-normalized read counts for each promoter, we calculated the pairwise Euclidean distance between each pair of proteins. We found that Dsim Zeus is most distant from the other two proteins, which is consistent with the pattern of molecular evolution (Figure 1). Bars represent 95% bootstrap confidence intervals. Figure S13: Histogram of random motifs’ F Statistics. In order to verify that the CAZAM showed an especially unusual pattern of evolution, we generated 100 random motifs and checked their pattern of evolution in nine species, computing an F Statistic, as described in the Supplementary Methods. The blue histogram shows the random motifs’ F Statistic values, while the black line labeled “Actual” shows the F statistic computed for the CAZAM, which is much greater. Table S1: Sequencing statistics. For each replicate, we show the total number of reads and total number of uniquely mapping reads. In all cases, there was sufficient depth to produce accurate and highly replicable (see Supplementary Figure S10) measurements of Chip signal. Table S2: CAZAM motif matches in different genomes. For each species (with reference genome versions on the right), we have the total number of perfect matches to the CAZAM motif, as well as the total number of base pairs searched and the motifs/10 kbp. Species with Zeus are shaded yellow; species without are shaded green. Below, we computed the total motifs per 10 kbp for species with and without Zeus. In Sheet Two, we show the same data, but only for the X chromosome. Most species’ genomes were sufficiently fragmented, so that there was no identifiable X chromosome, so we limited ourselves to species with previously identified X chromosomes. In Sheet Three, we show the same data as above, but for the autosomes (everything but the X chromosome). Table S3: Modified McDonald–Kreitman test considering SNPs of minor allele frequency greater than 0.05. We display polymorphism and divergence data for motifs and flanks. The first two columns show the species in which motifs were located within and mapped to. The next four columns show divergence (within, dN; and flanking, dS) and polymorphism (within, pN; flanking, pS). The next column shows the estimated value of α, the proportion of adaptive substitutions, computed as (1 − (dS * pN)/(dN * pS)). The next two columns show low and high 99% bootstrapped confidence intervals on α, and the G test *p*-value for the reported ratios of polymorphism and divergence. In Sheet Two, the same data are presented, but stratified by whether the motif falls within 100 base pairs of an annotated exon in *Drosophila melanogaster*. In Sheet Three, the same data are presented, but stratified by whether the motif falls on the X chromosome or an autosome after mapping into *melanogaster*. Table S4: Modified McDonald–Kreitman test considering SNPs of minor allele frequency greater than 0.1. As in Supplementary Table S3, but considering SNPs of MAF greater than 0.1. Table S5: Modified McDonald–Kreitman test incorporating indels. As in Supplementary Table S3, but incorporating indels and applying no minor allele frequency cutoff. Table S6: Modified McDonald–Kreitman test using nearby synonymous variation. As in Supplementary Table S3, but using polymorphism and divergence at synonymous codons within 1 kb as a neutral reference, instead of flanking sites. We report only the overall results, not parsing by sequence type or chromosome. Table S7: Modified McDonald–Kreitman test using random motifs. As in Supplementary Table S3, but using randomly generated permutations of the CAZAM motif. The chosen permutation is in the first column, while the species the motif was located within and mapped from is in the second. We report only overall results, not chromosome- or sequence-specific tabulations. Table S8: Alignment statistics across control and *Zeus* knockout *Drosophila melanogaster* testis RNA-Seq samples. Table S9: Table of 661 differentially expressed genes between control and *Zeus* knockout testis RNA-Seq samples. Table S10: Gene ontology (GO) analysis results. On the left, GO analysis results for 330 downregulated genes in *Zeus* knockout testes samples relative to control testes. On the right, GO analysis for 331 upregulated genes in *Zeus* knockout testes relative to control testes
