# Supplementary material for: Rapid Cis–Trans Coevolution Driven by a Novel Gene Retroposed from a Eukaryotic Conserved CCR4–NOT Component in Drosophila
Source: Genes (Basel). 2021 Dec 26;13(1):57. doi: 10.3390/genes13010057 (PMC8774992; doi:10.3390/genes13010057)

Supplementary Figure S1

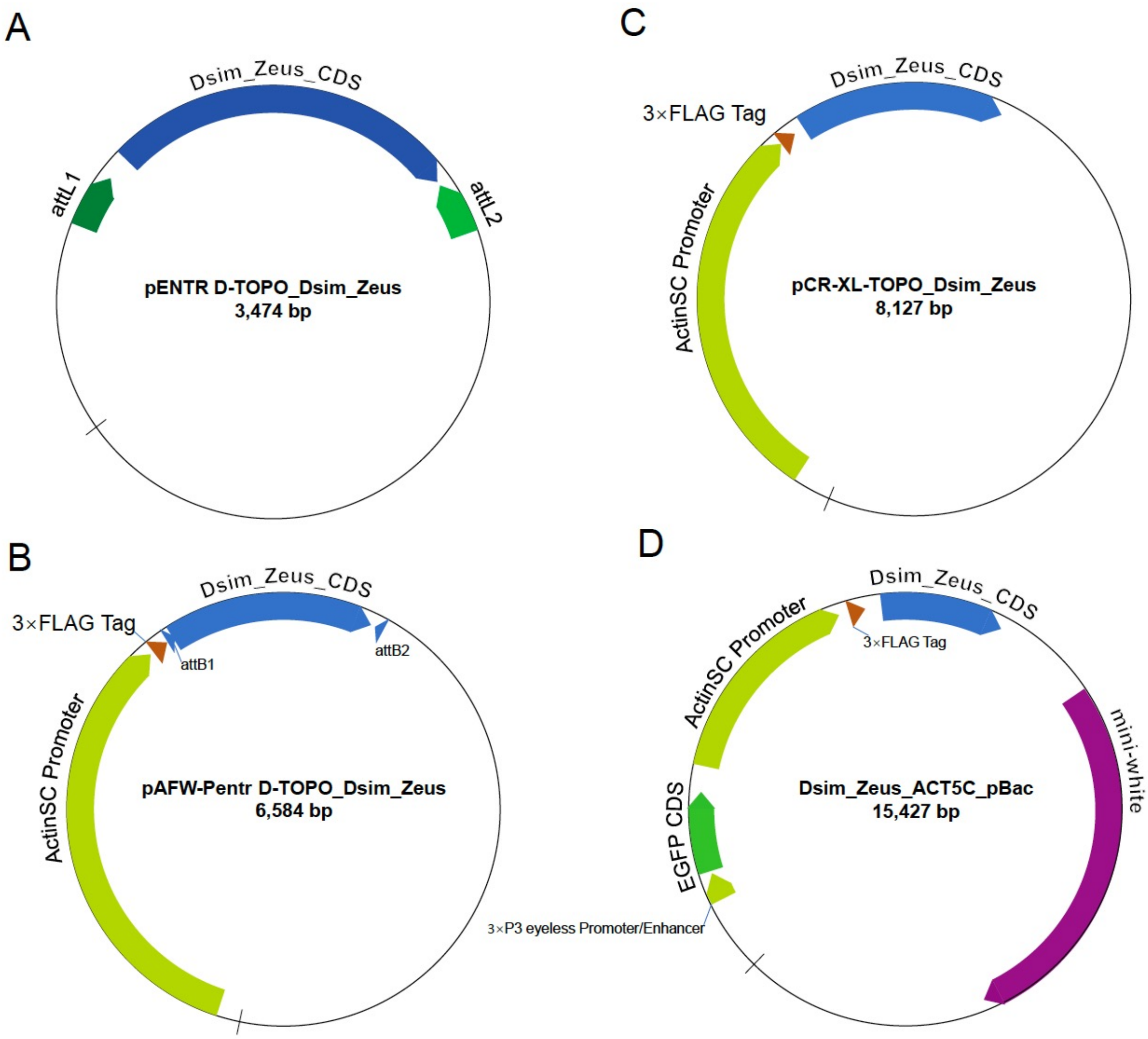

Supplementary Figure S2

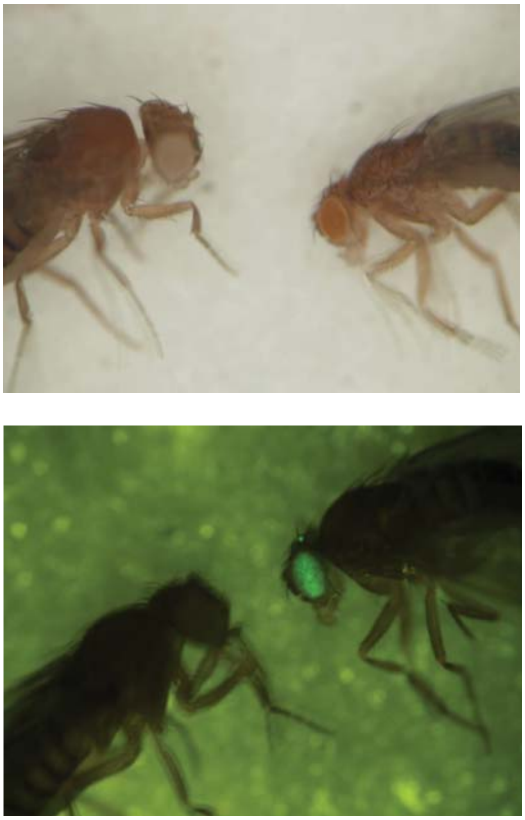

Supplementary Figure S3

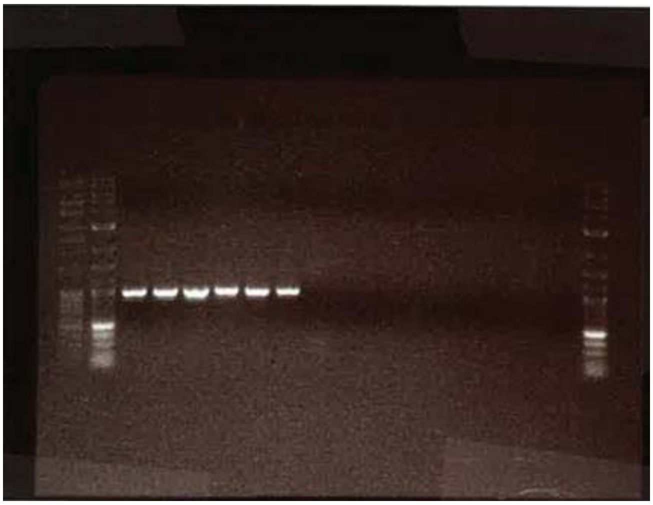

Supplementary Figure S4

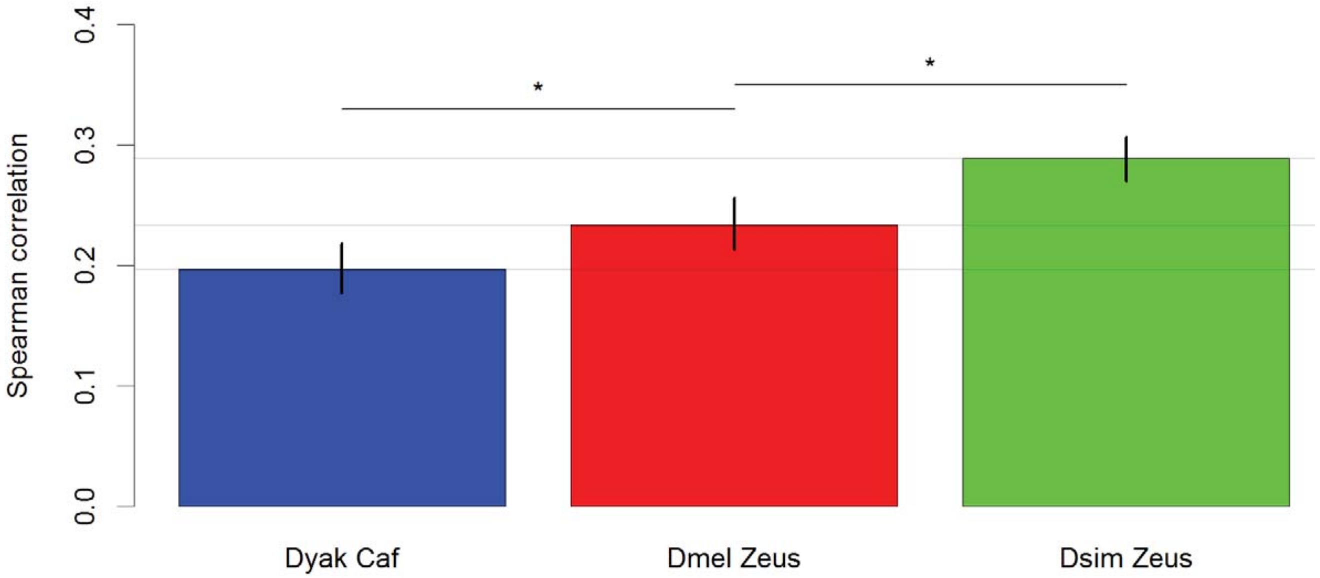

Supplementary Figure S5

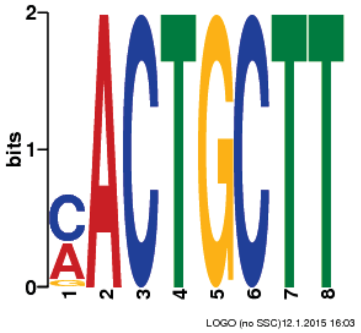

Dmel Zeus

Supplementary Figure S6

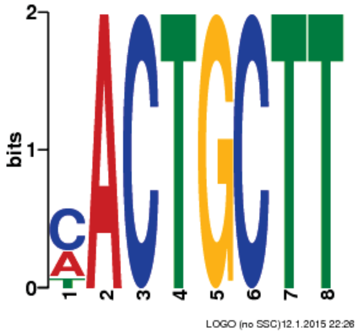

Dsim Zeus

Supplementary Figure S7

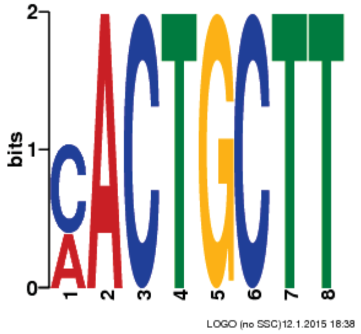

Dyak Caf

Supplementary Figure S8

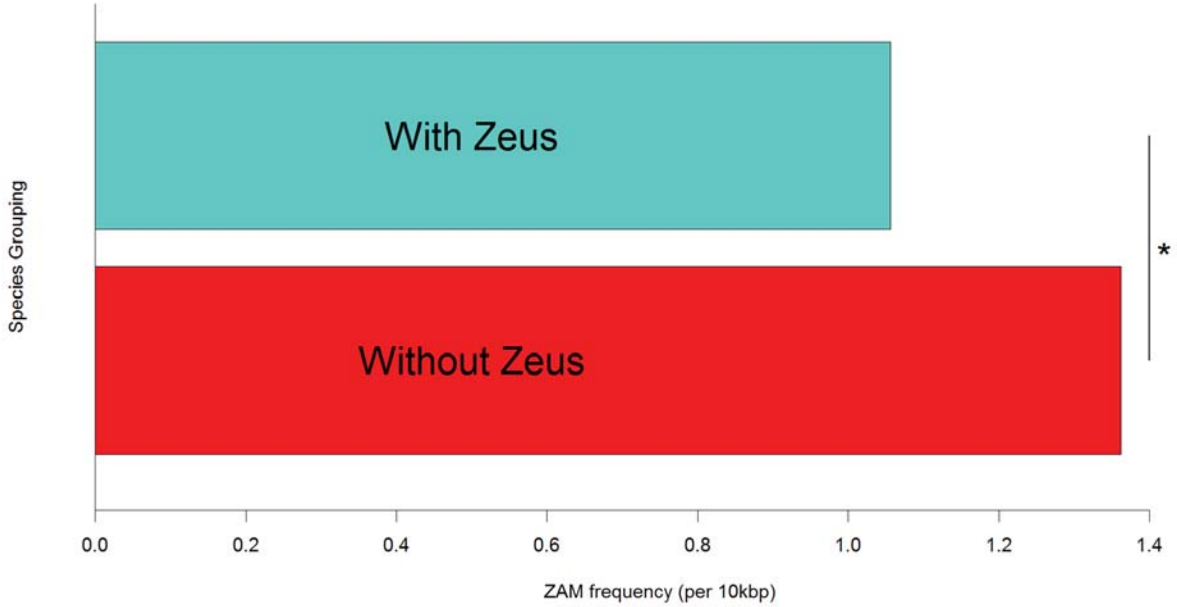

Supplementary Figure S9

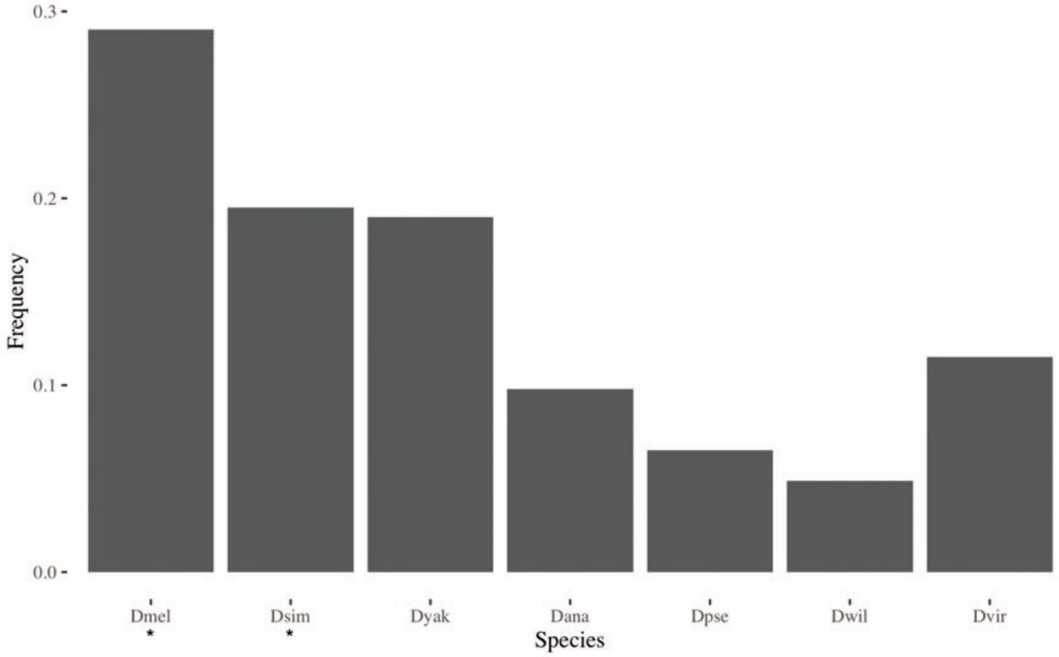

Supplementary Figure S10

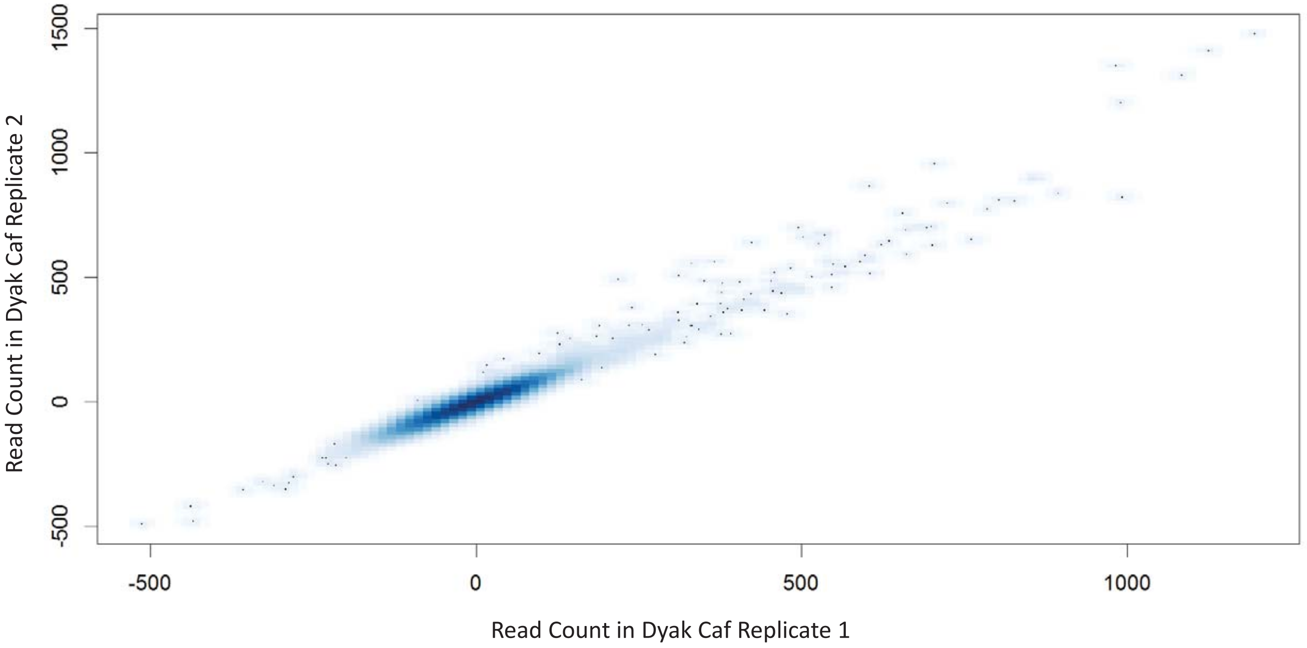

Supplementary Figure S11

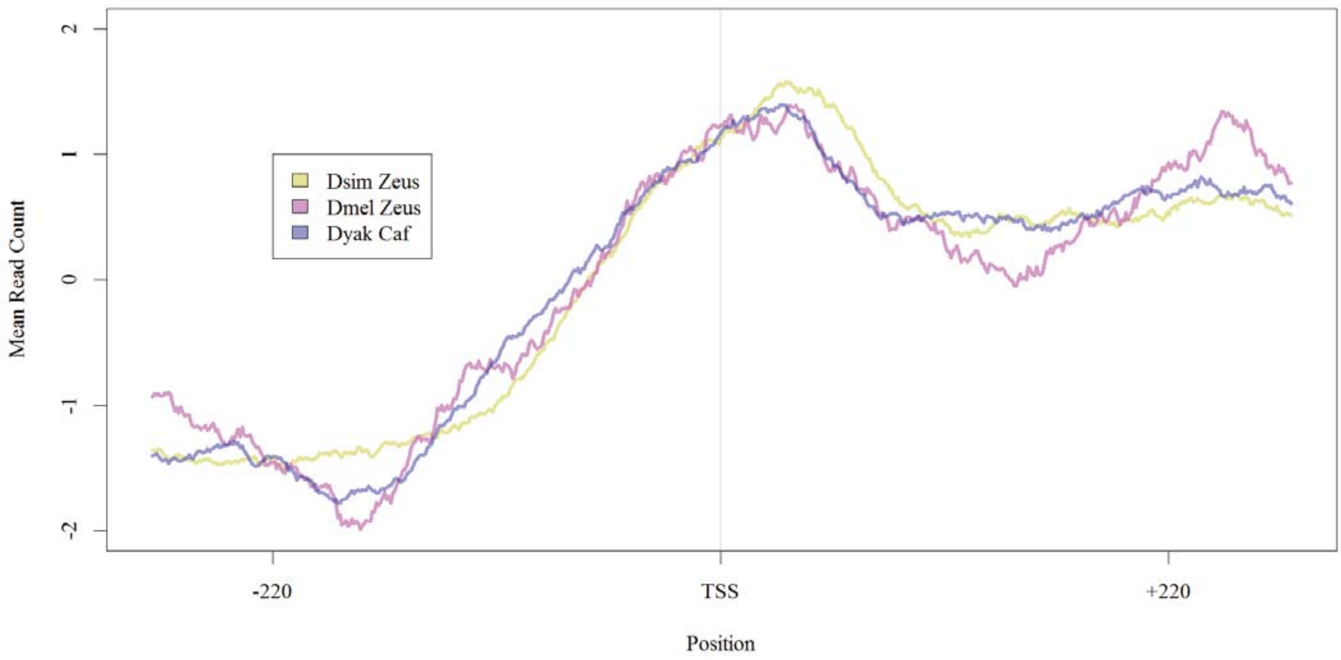

Supplementary Figure S12

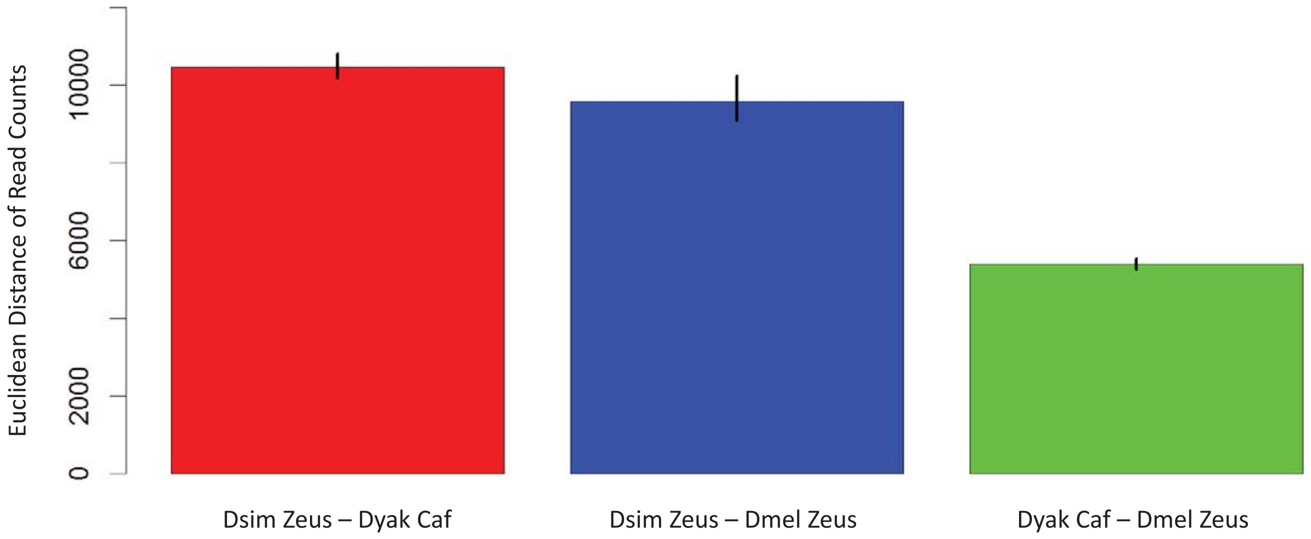

Supplementary Figure S13

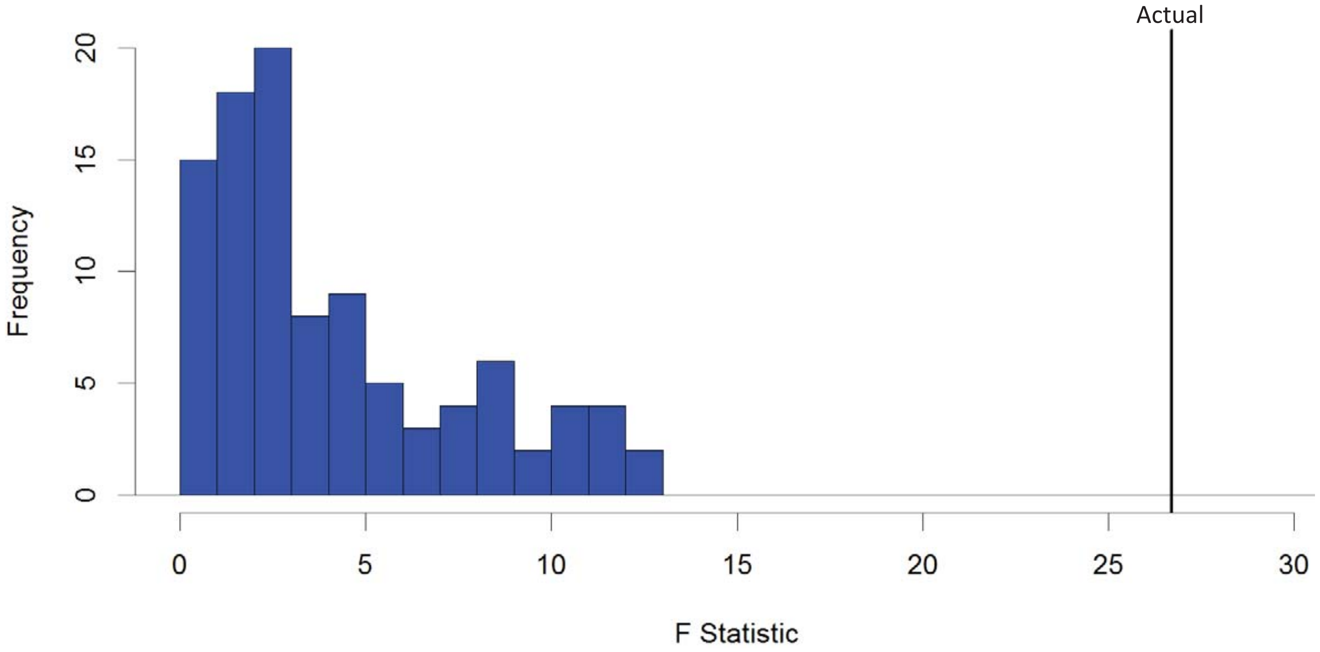

Supplement: Supplementary file 1 [file genes-13-00057-s001.zip › SupplementaryFiguresS1-S13_Submitted.pdf]
